# Impact of Substance Use Disorder Curriculum and X-Waiver Training on Emergency Medicine Residents

**DOI:** 10.5811/westjem.50864

**Published:** 2026-06-16

**Authors:** Chigozie Chukwunyere, Jessica Heil, Matthew Salzman, Rachel Haroz

**Affiliations:** * Cooper University Health Care, Center for Healing, Division of Addiction Medicine, Camden, New Jersey; † Cooper University Health Care, Department of Emergency Medicine, Division of Addiction Medicine and Medical Toxicology, Camden, New Jersey

## Abstract

**Introduction:**

We incorporated an 8-hour standardized substance use disorder (SUD) curriculum and X-waiver training into our emergency medicine (EM) residency. We sought to assess whether the implementation of the standardized SUD curriculum and X-waiver training affected graduated EM residents’ comfort with treating SUD and prescribing practices, as well as their view of their future career paths.

**Methods:**

Physicians who completed their EM residency at our hospital from 2016–2022 were invited to complete the survey in 2023. The primary outcome measure was the proportion of surveyed graduates who reported comfort and confidence in treating patients with SUD, measured by affirmative responses to survey items. The secondary outcome measure was the proportion of surveyed graduates reporting that participation in the SUD curriculum and X-waiver training during residency influenced their career plans, measured by affirmative responses to career-related survey items. For each outcome measure, we compared percentages of affirmative responses between physicians who completed residency prior to and after the implementation of the SUD curriculum and X-waiver training.

**Results:**

Among 81 graduated EM residents invited to complete the survey, 63 (78.8%) responded. We grouped them based on whether they had completed residency prior to the addition of the standardized SUD curriculum (pre-curriculum, 2016–2017) or after (post-curriculum, 2018–2022). Of the 63 participants, 17 were pre- and 46 were post-curriculum residents. Of the pre-curriculum residents, 58.8% prescribed buprenorphine in their practice after residency, compared to 76.1% of the post-curriculum residents (Fisher exact test, *P* = .22, odds ratio [OR] 0.46, 95% confidence interval, 0.12–1.76). Of the post-curriculum graduates, 93.5% noted that they were better prepared to treat patients with SUDs than their peers compared to 88.2% of the pre-curriculum graduates (Fisher exact test, *P* = .605, OR .529, 95% CI, 0.055–6.909), and 69.6% believed the training impacted their careers professionally and increased their interest in helping patients with opioid use disorder.

**Conclusion:**

Overall, a greater proportion of EM graduates who had been X waiver-trained in a substance use disorder curriculm during residency training at our institution prescribed buprenorphine to their patients than those who had not undergone specific training. Although the X-waiver is no longer a requirement, our SUD curriculum and training remain relevant for buprenorphine prescribing and may offer some opportunities for EM residents to develop their careers.

## INTRODUCTION

The opioid epidemic remains a public health crisis, resulting in 81,083 opioid-related overdose deaths in 2023 in the United States.^1^ Buprenorphine, a partial mu-receptor opioid agonist, has been approved as treatment for opioid use disorder (OUD) since 2002; however, it is underused in the United States, with only 25% of adults with OUD receiving this effective medication.^2^, ^3^ Emergency departments (ED) continue to be a crucial interface for establishing access to care and decreasing mortality.^4^, ^5^ Thus, training emergency medicine (EM) residents to treat OUD should be considered critical in improving low-barrier access to evidence-based substance use care.

The Accreditation Council for Graduate Medical Education requires all graduate medical education programs to incorporate addiction training into their curricula.^6^ Residency programs that have incorporated addiction training have shown tremendous success in improving knowledge and reducing the stigma of OUD treatment.^7^ In one study, 79% of internal medicine residents felt more prepared to treat patients with OUD following didactic training.^8^ Yet a substance use disorder (SUD) curriculum, specifically one focused on OUD education and training, has not been standardized or incorporated into EM resident education nationally.

In 2016, our institution began initiating and prescribing buprenorphine in the ED. Subsequently, in 2017 a SUD curriculum was formalized for both faculty and residents.^9^ Interested residents from our institution, along with emergency physicians, contributed to the curriculum development. We successfully implemented an 8-hour SUD curriculum and X-waiver training for residents starting in 2018. The X-waiver training focused on OUD treatment, which was required for physicians to prescribe buprenorphine. The curriculum also featured formal SUD lectures, clinical exposure at an addiction-medicine specialty clinic, and a pre- and post-curriculum assessment. Before 2018, residents received SUD training solely through clinical experience and a limited lecture series. Although the X-waiver regulatory program itself no longer exists, our EM residency’s SUD curriculum and OUD training remain relevant for meeting current training requirements and facilitating buprenorphine prescribing.

In this study we sought to examine the impact of this intervention on graduated residents’ practice and their comfort with treating patients with SUD. Additionally, we aimed to explore the perceptions of the SUD curriculum and mandatory X-waiver training on graduated residents’ career trajectories.

We hypothesize that the standardized SUD curriculum and mandatory X-waiver training led to more professional opportunities and greater confidence in treating OUD among graduated EM residents.

## METHODS

### Study Setting

The hospital from which we recruited survey participants is a large, tertiary-referral and trauma center. The ED sees approximately 82,000 visits a year and hosts a 3-year residency as well as multiple fellowships.

### Survey Content and Administration

The survey was developed through a collaborative process, leveraging the expertise of two addiction medicine and EM board-certified physicians who designed the curriculum at our institution. The survey asked about graduates’ current careers and whether the SUD curriculum has impacted their prescribing practices, improved confidence in treating SUD, and impacted their career. One open-ended question asked the following: “If your career was impacted by education and training during your residency in SUD and medications for opioid use disorder/X-waiver training, please explain below.” The survey and the open-ended responses are available as supplemental material.

**Population Health Research Capsule**

What do we already know about this issue?

*Residency programs with substance use disorder (SUD) curricula have been shown to increase residents’ comfort with treating patients with SUD*.

What was the research question?

*Did our SUD curriculum and X-waiver training improve emergency medicine (EM) residents’ SUD confidence and career trajectories after they graduated?*

What was the major finding of the study?

*58.6% of pre- vs 76.1% of post-curriculum residents prescribed buprenorphine after graduating (odds ratio 0.46, 95% CI, 0.12–1.76; P =.22)*.

How does this improve population health?

*Incorporating a standardized SUD curriculum and opioid use disorder training into EM residency promotes evidence-based substance use care*.

The EM residency maintains a running list of contact information from physicians who completed the residency between 2016–2022. In 2023, recruitment emails with a unique Research Electronic Data Capture (REDCap) survey link were sent to graduates, ensuring identifiable information remained separate from responses.^10^, ^11^ Written informed consent was used, and consent was obtained after participants continued from the consent form to the survey. We excluded EM residents if they did not complete the survey or had yet to graduate from residency. The participants were compensated for completing the survey with a $25 gift card. The study was approved and deemed exempt by the hospital institutional review board.

### Data Analysis

We conducted descriptive statistics using Microsoft Excel v2505 (Microsoft Corp, Redmond, WA). Statistical analysis was completed using RStudio v4.4.2 (Posit, PBC, Boston, MA).

## RESULTS

Of the 80 graduated residents invited to participate, 63 (78.8%) responded and completed the survey (Table 1). Most graduates reported working in an urban setting (58.7%) within a community hospital (60.3%).

Overall, 71% of the graduates reported currently prescribing buprenorphine, and 92% of them had received their X-waiver (Table 2). Half (50.8%) of the graduates were working within an ED that had a formal protocol for buprenorphine initiation or dosing; of those graduates, 12.5% had helped create that protocol.

To determine the impact of a formalized curriculum on the graduates, we separated the graduated residents into two groups: before the standardized SUD curriculum was added to the program (pre-curriculum, 2016–2017) and after the curriculum was in place (post-curriculum, 2018–2022). Of the 63 graduates who responded to the survey, 17 had been pre-curriculum and 46 post-curriculum residents. We found that 58.8% of pre-curriculum graduates prescribe buprenorphine, while 76.1% of the post-curriculum graduates prescribe buprenorphine. There was no statistically significant difference between the pre-curriculum and post-curriculum groups in their current buprenorphine prescribing practice (Fisher exact test, *P* = .22, odds ratio [OR] 0.46, 95% confidence interval, 0.12–1.76).

Nearly all graduates (95.3%) indicated that both clinical experience and didactic/X-waiver training had prepared them to treat SUD patients (Fisher exact test, *P* = 1.00, OR 0, 95% CI, 0–1167.81). There was a strong association between SUD clinical training impact and SUD education (Fisher exact test, *P* < .001, OR infinity, 95% CI, 6.88-infinity). Among graduates who indicated that their SUD clinical training impacted their career choice, 100% reported that their SUD education also impacted their career. Further, 50% of graduates who indicated that their SUD education impacted their career choice also did not indicate that their SUD clinical training impacted their career.

Of the post-curriculum graduates, 93.5% felt that they were better prepared to treat patients with SUDs than their colleagues at their current workplace, compared to 88.2% of pre-curriculum graduates (Fisher exact test, *P* = .605, OR .529, 95% CI, 0.055–6.909). Lastly, for the open-ended question, 69.6% of post-curriculum graduates felt the training had affected their careers by allowing them to grow professionally and increase their interest in the OUD population (Table S1).

## DISCUSSION

Our results suggest that EM residents who completed a formal SUD curriculum and X-waiver training credited that training with impacting their prescribing practices and to a lesser degree their career development.

It is worth noting that in 2022, the U.S. Congress eliminated the X-waiver requirement for buprenorphine prescribing from the Mainstreaming Addiction Treatment Act.^12^, ^13^ However, a separate act required that physicians satisfy at least one of multiple conditions to prescribe buprenorphine, including the completion of a residency curriculum with a minimum 8-hour OUD training.^13^ The X-waiver elimination presents an opportunity to re-examine how our SUD curriculum is structured and delivered to EM residents. Data show that a large proportion of waivered clinicians in the United States do not prescribe buprenorphine.^14^ Kim and Samuels highlighted clinician discomfort with dispensing and prescribing buprenorphine as a barrier to ED buprenorphine.^15^

Research suggests that integrating SUD education into clinician training is an effective strategy for enhancing physician comfort, bridging knowledge gaps, and increasing buprenorphine prescribing.^16^ Our findings support these claims; graduates who were post-curriculum residents reported feeling knowledgeable, confident, and comfortable with patients after completing the SUD curriculum and X-waiver training, and a large majority of these graduates tended to prescribe buprenorphine to their current patients. Although the buprenorphine-prescribing practices between the pre- and post-curriculum groups were not statistically significant, there was a slightly higher percentage of the post-curriculum group who prescribed buprenorphine compared to the pre-curriculum group. This was despite residents being X-waivered either during residency (post-curriculum) or afterward (pre-curriculum residents).

Additionally, we examined the impact of the SUD curriculum and X-waiver training on EM residents’ career development. In the open-ended portion of our survey, some post-curriculum residents noted that their OUD knowledge provided more opportunities to educate their colleagues about OUD treatment, choose addiction-related fellowships, and secure leadership positions. Adding the open-ended question allowed us to probe deeper into the responses and find more subtle indications of career advancement and development. The survey responses also revealed that some of our residency graduates acted as physician champions for buprenorphine usage at their current jobs, which likely improves care for their patients with OUD and advances their colleagues’ understanding of OUD.

Interestingly, few post-curriculum residents answered “Yes” during the Yes/No portion of the survey when asked whether their SUD training had impacted where they chose to work (19.6%) or their SUD education and training impacted their career (37.0%). This could be due to varying interpretations of the word “impact” and, as a result, residents may not have counted certain informal opportunities as influencing their career, leading to more “No” responses. When we considered all residency graduates, however, we found that perceived influences of SUD education and training on career choice were closely linked, and educational exposure appeared to have a broader influence on career choice than SUD training.

Although we did not explicitly ask respondents whether they were generally satisfied with their current jobs, the literature suggests that the training may be associated with increased job satisfaction for these residency graduates.^17^–^20^ When physicians perceive themselves as providing high quality care to patients and are given services that facilitate their jobs, their professional satisfaction and satisfaction toward their patients increases.^17^–^20^

## LIMITATIONS

This study took place at a single institution. All EM residents at our academic, urban hospital were exposed to a larger-than-average SUD population and had access to a robust outpatient-referral capacity. This may limit the generalizability of the results. The survey that we sent out to graduates was not validated, as there were no validated surveys in the literature that captured the data we were interested in. We administered the survey in February 2023, around the same period that the X-waiver requirement was repealed. Some graduates may not have found the X-waiver component of the SUD curriculum to be relevant to their current practice, potentially impacting their responses. The institutions that the graduates currently work at could have also influenced their buprenorphine-prescribing patterns. Additionally, social desirability bias may have influenced how residents answered survey questions, as residents may have self-reported that they prescribe buprenorphine or have their X-waiver to align with perceived study expectations.

Finally, the pre-curriculum response numbers were smaller than post-curriculum response numbers. This may be related to the initiation of buprenorphine prescribing at our institution in 2016, and the subsequent implementation of the SUD curriculum and X-waiver training in 2018, resulting in a smaller pool of eligible pre-curriculum residents. Because our study had a low sample size, running statistics on the dataset resulted in sparse cell counts for test comparisons.

## CONCLUSION

This study suggests that incorporating a standardized substance use disorder curriculum and X-waiver training into EM residency may increase EM residents’ confidence and comfort in treating opioid use disorder and may offer some opportunities for career advancement. Our results demonstrate that residency training remains an essential opportunity to promote the implementation of evidence-based addiction treatment in the ED. Future work should examine graduated residents’ clinical practice and career trajectories in the post X-waiver era.

## Supplementary Information





## Figures and Tables

**Table 1:** Demographic characteristics of emergency medicine residents in a study assessing the effects of implementing a standardized substance use disorder curriculum and X-waiver training.

Survey Question	All Resident Groups	Pre-curriculum (2016–2017)	Post-curriculum (2018–2022)
Currently working in the emergency department			
Yes	59 (93.7)	15 (88.2)	44 (95.7)
No	4 (6.3)	2 (11.8)	2 (4.3)
Currently in a fellowship			
Yes	7 (11.1)	0 (0)	7 (15.2)
No	56 (88.9)	17 (100.0)	39 (84.8)
Primary location			
Rural	3 (4.8)	1 (5.9)	2 (4.3)
Suburban	23 (36.5)	8 (47.1)	15 (32.6)
Urban	37 (58.7)	8 (47.1)	29 (63.0)
Type of Hospital			
Academic	24 (38.1)	5 (29.4)	19 (41.3)
Community	38 (60.3)	11 (64.7)	27 (58.7)
Federal (Veterans Affairs)	1 (1.6)	1 (5.9)	0 (0)
Receive substance use disorder lectures during didactic time			
Yes	61 (96.8)	16 (94.1)	45 (97.8)
No	2 (3.2)	1 (5.9)	1 (2.2)
Did you participate in a 4-hour (1/2 and 1/2) X-waiver training during residency?			
Yes	50 (79.4)	6 (35.3)	44 (95.7)
No	13 (20.6)	11 (64.7)	2 (4.3)
As a resident, did you and your attending prescribe and refer patients to follow-up during your residency?			
Yes	55 (87.3)	10 (58.8)	45 (97.8)
No	8 (12.7)	7 (41.2)	1 (2.2)

**Table 2:** Survey data related to prescribing practices, comfort with patients, and career trajectories of emergency medicine graduates in a study assessing the effects of implementing a standardized substance use disorder curriculum and X-waiver training

Survey Question	All Resident Groups	Pre-curriculum (2016–2017)	Post-curriculum (2018–2022)
Did you obtain your X-waiver?			
Yes	58 (92.1)	14 (82.4)	44 (95.7)
No	5 (7.9)	3 (17.6)	2 (4.3)
Does your emergency department have a formal protocol for buprenorphine initiation or dosing?			
Yes	32 (50.8)	6 (35.3)	26 (56.5)
No	31 (49.2)	11 (64.7)	20 (43.5)
If yes, did you help create this protocol?			
Yes	4 (6.3)	0 (0)	4 (8.7)
No	28 (44.4)	6 (35.3)	22 (47.8)
(blank)	31 (49.2)	11 (64.7)	20 (43.5)
Do you prescribe buprenorphine to patients now?			
Yes	45 (71.4)	10 (58.8)	35 (76.1)
No	18 (28.6)	7 (41.2)	11 (23.9)
Do other physicians, nurses, or hospital staff utilize your knowledge in this area to help them with patient care?			
Yes	48 (76.2)	12 (70.6)	36 (78.3)
No	15 (23.8)	5 (29.4)	10 (21.7)
Do you think your experience treating patients with opioid use disorder and substance use disorder during residency affected your approach to patients now?			
Yes	62 (98.4)	17 (100.0)	45 (97.8)
No	1 (1.6)	0 (0.0)	1 (2.2)
Did the didactics and X-waiver training prepare you to treat patients with substance use disorder?			
Yes	61 (96.8)	17 (100.0)	44 (95.7)
No	2 (3.2)	0 (0.0)	2 (4.3)
Do you feel that you were better prepared to treat patients with substance use disorders than your colleagues where you work now?			
Yes	58 (92.1)	15 (88.2)	43 (93.5)
No	5 (7.9)	2 (11.8)	3 (6.5)
Has your substance use disorder training impacted where you chose to work and other career choices?			
Yes	10 (15.9)	1 (5.9)	9 (19.6)
No	53 (84.1)	16 (94.1)	37 (80.4)
Has your education and training in substance use disorder and medications for opioid use disorder/X-waiver training impacted your career? (i.e., helped you get a job, more opportunities, advancement)			
Yes	21 (33.3)	4 (23.5)	17 (37.0)
No	42 (66.7)	13 (76.5)	29 (63.0)
