# Supplementary material for: Impact of Substance Use Disorder Curriculum and X-Waiver Training on Emergency Medicine Residents
Source: West J Emerg Med. 2026 Jun 16;27(4):880–5. doi: 10.5811/westjem.50864 (PMC13436645; doi:10.5811/westjem.50864)
Supplement: Supplementary file 1 [file wjem-27-880-s001.docx]

**Survey Instrument**

1. When did you graduate from Cooper University Health care's emergency medicine residency?
   a. 2016
   b. 2017
   c. 2018
   d. 2019
   e. 2020
   f. 2021
   g. 2022
2. Are you currently working in an emergency department?
   a. Yes (Even if in a fellowship or working part-time or per diem)
   b. No
   c. Currently not working
3. If you aren't currently working in an emergency department or fellowship, what are you doing?
   a. _____________________________
4. Are you currently working full-time, part-time, per diem, or currently not working?
   a. full-time
   b. part-time
   c. per-diem
5. Are you currently in a fellowship?
   a. Yes
   b. No
6. If you are in a fellowship, what kind of program are you a part of?
   a. Addiction medicine
   b. Ultrasound
   c. Emergency medical services
   d. Trauma and/or Critical Care
   e. Toxicology
   f. Wilderness Medicine
   g. Sports Medicine
   h. Research
   i. Pediatric Emergency Medicine
   j. Medical Education, Academics/Faculty Development
   k. International Emergency Medicine
   l. Hyperbaric and Undersea Medicine
   m. Hospice and Palliative Medicine
   n. Administration
   o. Geriatrics
   p. Other
7. Describe the other type of fellowship program you are a part of currently.
   a. _____________________________
8. How would you describe the primary location where you currently practice medicine?
   a. Rural
   b. Urban
   c. Suburban
9. How would you describe the primary location where you currently practice medicine?
   a. Academic
   b. Community
   c. Federal (VA)
10. Did you receive substance use disorder lectures during your didactic time at Cooper?
    a. Yes
    b. No
11. Did you participate in a 4-hour (1/2 and 1/2) X-waiver training during residency?
    a. Yes
    b. No
12. As a resident, did you and your attending prescribe and refer patients to follow-up during your residency?
    a. Yes
    b. No
13. Did you obtain your X-waiver before or after graduation?
    a. Before graduation
    b. After graduation
    c. I didn't receive my X-waiver
14. Does your ED have a formal protocol for buprenorphine initiation or dosing?
    a. Yes
    b. No
15. If yes, did you help create this protocol?
    a. Yes
    b. No
16. Do you prescribe buprenorphine to patients now?
    a. Yes
    b. No
17. If you do prescribe buprenorphine to patients, how many prescriptions have you completed?
    a. 1-10
    b. 10-50
    c. 50-100
    d. >100
18. Are you able to refer patients?
    a. Yes
    b. No
19. Do other physicians, nurses, or hospital staff utilize your knowledge in this area to help them with patient care?
    a. Yes
    b. No
20. Do you think your experience treating patients with opioid use disorder and substance use disorder during residency affected your approach to patients now?
    a. Yes
    b. No
21. Did the didactics and X-waiver training prepare you to treat patients with substance use disorder?
    a. Yes
    b. No
22. Do you feel that you were better prepared to treat patients with substance use disorders than your colleagues where you work now?
    a. Yes
    b. No
23. Has your SUD training impacted where you chose to work and other career choices?
    a. Yes
    b. No
24. Has your education and training at Cooper in SUD and medications for opioid use disorder/X-waiver training impacted your career? (i.e. helped you get a job, more opportunities, advancement)
    a. Yes
    b. No
25. If your career was impacted by education and training at Cooper in SUD and medications for opioid use disorder/X-waiver training, please explain below.
    a. _____________________________

*VA*, Veterans Affairs; *ED*, emergency department; *SUD,* substance use disorder
