# Supplementary material for: Impact of Substance Use Disorder Curriculum and X-Waiver Training on Emergency Medicine Residents
Source: West J Emerg Med. 2026 Jun 16;27(4):880–5. doi: 10.5811/westjem.50864 (PMC13436645; doi:10.5811/westjem.50864)
Supplement: Supplementary file 2 [file wjem-27-880-s002.docx]

**Table S1.** Survey responses of emergency medicine residents to the open-ended field: “If your career was impacted by education and training during your residency in substance use disorder and medications for opioid use disorder/X-waiver training, please explain below,”.

Better prepared to treat these patients

I received excellent substance abuse training in residency at Cooper. As result, I felt competent and confident starting patients w/ OUD on MAT. When I started as a new attending in 2017, I was leader of the pack in terms of MAT amongst my new physician colleagues. I helped teach and train other physicians in MAT. Thanks to Dr Haroz and the crew at Cooper!

I think we had some SUD training but suspect later classes have had more as bupe became more widely prescribed. I should and would like to be more comfortable with prescribing

N/a

Training in a culture at Cooper where Bupe was beginning to be mainstream, I feel very comfortable using it now and have been able to teach a lot of my colleagues about it at several clinical sites.

Better able to provide care to SUD patients, but hasn't directly affected my career

Appreciated the sud clinic

NA

N/a

I am much more comfortable treated both overdoses and opioid use disorder patients in my emergency department, and convincing them to follow up with our resources that we provide. I have had repeat patients who come back to my ED due to the fair, extensive, and non-judgmental care they receive.

I was "the guy" people asked about X-Waivers and how I prescribe suboxone. But it didn't really impact my career directly.

N/A

it was helpful

When working for MedStar, I was often the only doc who could Rx for bupe. I always offered it to people, eventually one SUD peer coach would always seek me out when he knew people needed a rx. I was always surprised how much less opiate use there was in the DC metro area compared to camden. I do think that training at cooper was more of an important piece of shaping my rx of bupe than the actual training didactic. Learned more from Matt and Rachel than anything else. I was happy I had the certification and I am glad the govt finally stepped up to get rid of it. I work in Norristown, PA now and there is much less opiate use but I still write it from time to time and use it often in the ED

I feel comfortable treating and educating on SUD at my current job.

Na

I feel very comfortable initiating treatment and find that many colleagues who did not receive similar training in residency do not have the necessary foundational knowledge. Shout out to Rachel Haroz and Matt Salzman who are pioneers in the field of addiction medicine and specifically OUD.

Na

Definitely more knowledgeable than others in treating, but also frustrating the barriers that are encountered at my facility and/or the lack of education- including with prescribing while patients are in the department/escalating doses (pharmacy resistance). Of note, protocol at my facility was partially adapted by a former Cooper resident several years ago. Setting up for followup is less reliable than at Cooper, but feel comfortable prescribing upon discharge.

It is not a huge part of my current role but the training and education was valuable

I definitely feel more comfortable initiating and prescribing buprenorphine to patients presenting in opioid withdrawal or with hx of opioid use disorder who are interested in buprenorphine.

My career was more influenced simply by exposure to Bup/MAT in treatment of SUD in residency. Its more like I understood it existed and what is was rather than how to apply it. During my senior year Matt and Rachel were just starting to get their clinic going, and I feel Bup in the ED was still a new thing. My new job in Alaska influenced me more to get training as it was an area of focus at our neighboring hospital in Anchorage and was an educational goal for both Alaska ACEP and Washington ACEP when I moved up here.

n/a

NA

Currently in an Addiction Medicine Fellowship.

N/A

While I don't frequently prescribe MAT due to lack of patient follow up opportunities, I have more education than my colleagues when it comes to the pathophysiology of SUD which helps me treat patients appropriately in my clinical setting.

I was asked to help with the group creating the bupe initiation protocol since it was known that I had great SUD training coming from Cooper. It was mentioned on my initial interview for an academic position.

I have a more thorough understanding and insight of SUD and my approach to treating patients. Although I was heavily involved in prehospital subuxone which probably contributed more to this than my co residents

I worked at urban level 1 trauma right out of residency for 4 years. I prescribed suboxone frequently. Often had older colleagues ask about how to use it or ask for assistance in prescribing for appropriate patients if they had not completed the training. Still helpful in my community / academic hybrid suburban department I am working in now. Still see plenty of OUD and still think about harm reduction and prescription of bup when appropriate. I tell my students / residents, you really get advanced education (experiential and didactic) in OUD training in Camden. Seems like I have a much higher comfort level with all aspects of OUD and ED management than many of my colleagues.

N/a

I do believe that I have a stronger knowledge base than my colleagues regarding the treatment for OUD however I have found barriers in the suburban setting that I work in. I have not prescribed bupe to my patients because I do not believe we have good established outpatient follow up for them to continue to get their prescription and follow up.

It helped me take care of patients better who present to the ER with substance abuse.

I feel better prepared to treat patients with opioid use disorder, I feel that I am less judgemental, more willing to prescribe bupe, and more confident in my ability to treat overdoses and other comorbidities of drug use.

My experience at cooper EM residency made me confident in prescribing buprenorphine- including induction, maintenance. The x-waiver training should be offered in the beginning of every year for new residents- it' was incredibly helpful.

My answers should be taken with the caveat that I no longer work in the ED. So when you ask if I work in the "community, ""academics," or "Federal (VA)" I really work in none of these--- and this applies to several questions. But overall my experience with the X Waiver has helped me understand the healthcare system better and how regulations can supersede commonsense. BUt--- sometimes you need to "play the game" when piloting a new initiative, and patients were served well in the long-run...

I learned to take care of a vulnerable patient population and it has impacted how I view them.

Has not impacted me in terms of opportunities but I feel more comfortable initiating conversations about MAT with patients with substance use disorder than a lot of my colleagues

N/A

More knowledgeable. Less afraid to prescribe.

I feel much more comfortable prescibing MAT and refering patients with SUD to follow up and feel confident that I am providing compassionate and evidence based medicine

I initially took my job in part because I could split my time in the ED and in an outpatient addiction medicine clinic which I did for some time. I became certified to give the x waiver training and trained providers both within and outside of my facility. With my colleagues I'm often asked to help with these patients and often have to clarify that I did not do an addiction fellowship but that I have a special interest in this area. Before the x waiver requirement was removed I was often asked to write this rx for other providers' patients and charge nurses would tell patients seeking help to come back while I was working a shift which they would look up for the patient!

N/a

SUD and X-waiver training was a unique requirement at Cooper that has given me the knowledge and skill set to treat and prescribe medications for SUD. The vast majority of my colleagues (even other recent graduates) do not have an X-waiver and do not have any training in SUD or MAT. This was a topic that I discussed confidently in job interviews and definitely gave me an advantage as an applicant.

I work at Cooper- but when I moonlight other places, I continue to obviously provide appropriate addiction care- which is rare in the half a dozen ED's I've moonlight at.

I definitely am more comfortable with prescribing suboxone than my colleagues. I find there are other attendings that do not have their xwavier training still.

n/a

I feel comfortable evaluating and treating patients with SUD. It seems I am much more comfortable with these patients than other colleagues.

Nad

n/a

I feel much more prepared than my colleagues to care for patients with substance use disorder.

Gave me tools to work with this patient population in the ED. Increased my interest in treating opioid use disorder and learning more, contributed to me choosing a fellowship in addiction medicine. My training empowered me to provide better care to my ED patients.

Focus on addiction medicine and working towards board certification via practice pathway

My training has helped me build my career in the ED as well as in public health, working with overdose systems of care and building networks for treatment for patient with OUD.

Have been able to take part in expanding the role of bupe/addiction medicine for EMS providers.

Has also contributed to personal job satisfaction as I feel that I now have some tools to help OUD patients.

I am comfortable prescribing suboxone and evaluating patients with SUD due to the didactics and training in residency.

My training challenges and provides balance to older physicians who approach addiction from grossly outdated perspectives.

N/A

I recently changed jobs in the past few months but am basing these answers on the job I was with for the past 4 years. We were one of the first groups to get the MAT training at cooper and it greatly impacted my practice. once I left cooper, I realized how rare this was at the time, the lack of education regarding MAT and the stigma that still existed. My hospital at the time did not know anything about suboxone or MAT and had nothing close to a protocol for prescribing this and referral. I worked closely with the directors and, with Rachel's help, was able to start an MAT program, write protocols and have set referral set up before the patient left the ED.

I have high degree of comfort prescribing suboxone. Follow up can be challenging. There is considerably less effort or interest in this where I currently practice given significantly less need from the local patient population.

*OUD*, opioid use disorder; *MAT*, medication-assisted treatment; *SUD*, substance use disorder; *ED*, emergency department; *Rx*, prescription; *ACEP*, American College of Emergency Physicians; *ER*, emergency room; *EM*, emergency medicine; *VA*, Veterans Affairs; *EMS*, emergency medical services
